# The global prevalence of ADHD in children and adolescents: a systematic review and meta-analysis

**DOI:** 10.1186/s13052-023-01456-1

**Published:** 2023-04-20

**Authors:** Nader Salari, Hooman Ghasemi, Nasrin Abdoli, Adibeh Rahmani, Mohammad Hossain Shiri, Amir Hossein Hashemian, Hakimeh Akbari, Masoud Mohammadi

**Affiliations:** 1 Department of Biostatistics, School of Health, Kermanshah University of Medical Sciences, Kermanshah, Iran; 2 Student Research Committee, Kermanshah University of Medical Sciences, Kermanshah, Iran; 3 Department of Psychiatry, Substance Abuse Prevention Research Center, Kermanshah University of Medical Sciences, Kermanshah, Iran; 4 Julius Wolff Institute (JWI) - Charité, Berlin, Germany; 5 Research Center for Environmental Determinants of Health (RCEDH), Health Institute, Kermanshah University of Medical Sciences, Kermanshah, Iran; 6 Cellular and Molecular Research Center, Gerash University of Medical Sciences, Gerash, Iran

**Keywords:** Attention deficit hyperactivity disorder, Children, Adolescents, Psychiatric disorder

## Abstract

**Background:**

Attention-Deficit / Hyperactivity Disorder is a developmental neurological disorder that has three basic characteristics: Attention Deficit, Hyperactivity, and impulsivity. This study aimed to investigate the prevalence of ADHD in children and adolescents.

**Methods:**

This investigation was carried out using the meta-analysis method under PRISMA guidelines. Until October 2020, the articles were gathered by scanning PubMed, Scopus, WOS, and Science Direct databases. The second version of Comprehensive Meta-Analysis software was used to run analyses after extracting data from chosen papers. At a significance level of 0.05, the I^2^ test was used to analyze study heterogeneity, and the Egger test was used to assess publication bias.

**Results:**

This analysis includes 61 cross-sectional research, with 53 research used to determine the prevalence of ADHD in children, 7.6% of 96,907 children aged 3 to 12 years had ADHD (95% confidence interval: 6.1–9.4%), and 5.6% of teenagers aged 12 to 18 years have ADHD (95% confidence interval: 4.8-7%). The prevalence of ADHD in children and adolescents according to the DSM-V criterion is also higher than previous diagnostic criteria, according to studies.

**Conclusion:**

The findings of this study based on meta-analysis show the high prevalence of attention deficit hyperactivity disorder (ADHD). The findings of this study demonstrate the importance of management and policy in the treatment and control of ADHD in children and adolescents.

**Supplementary Information:**

The online version contains supplementary material available at 10.1186/s13052-023-01456-1.

## Background

Attention Deficit Hyperactivity Disorder (ADHD) is one of the most frequent neurodevelopmental disorders in childhood and adolescence, which is typically first, diagnosed in childhood and often persists into adulthood. It is one of the most prevalent causes of children being sent to psychology and psychiatric clinics [1, 2]. Children with ADHD may struggle to focus, lack self-control and impulsive behaviors, or exhibit excessive activity [1–3]. Hyperactivity, one of the main symptoms of ADHD, can lead to a child’s psychological and social incompatibility at home, school, and community [2]. The fundamental aspect of Attention Deficit Hyperactivity Disorder ADHD, according to the Fifth Diagnostic and Statistical Manual of Mental Disorders (DSM5), is a set of behaviors that disturb social status. Increased motor activity in inappropriate contexts, excessive wobbling, finger play, and talkativeness are examples of these social circumstances, which can interfere with personal and educational life [3].

Attention deficit disorder affects children differently depending on their age and gender, and it is more common in boys than in girls [2]. The initial signs of hyperactivity are frequently hard to detect until a child reaches the age of four, and it is most evident in elementary school. In early adolescence, the condition normally follows a predictable pattern. Yet, studies demonstrate that the main characteristics of ADHD change with age, so regardless of classification, ADHD seems to reduce in adulthood. Throughout this age range, hyperactivity in particular, though still present, is less evident (Ramtekkar et al. 2010). As a result, a thorough history is required for the diagnosis of ADHD which then leads to the identification of specific symptoms [5].

Symptoms of ADHD are frequent in school-aged children and can last far into adulthood. In general, ADHD is usually characterized by impulsivity, attention deficit, and hyperactivity. A person with inattention may have trouble remaining on task, maintaining focus, and maintaining organization. A hyperactive person may appear to move around frequently or fidget excessively. An impulsive person may behave without thinking or struggle with self-control. [2–6]. Patients may be unable to pay close attention to detail or may be careless when performing tasks such as schoolwork, job, or other activities.

As previously mentioned, boys are more likely to manifest ADHD symptoms [6] with three times more likely to receive an ADHD diagnosis than girls [9]. Based on a study by Rucklidge, there are significant differences in how the condition reveals in boys and girls. Boys with ADHD usually show externalized symptoms, such as running and impulsivity. Girls with ADHD, on the other hand, typically show internalized symptoms [9]. These symptoms include inattentiveness and low self-esteem. Boys also tend to be more physically aggressive and externalize their frustrations, while girls tend to be more verbally aggressive and turn their pain and anger inward, putting them at an increased risk for depression, anxiety, and eating disorders [9].ADHD in children is caused by several factors, including genetics and environment, premature birth, preeclampsia, hypertension, overweight, and obesity in pregnant women, as well as maternal smoking exposure [7, 8]. Family history studies have also shown that drug abuse plays a significant role in the development of ADHD [9] Identifying whether a child has ADHD is a multi-step process. There is no single test that can detect ADHD. To diagnose ADHD a checklist for rating the symptoms is provided and a history of the child from parents, teachers, and sometimes, the child is taken [3–9].

Guardiola et al. carried out a study in which they assessed the prevalence of ADHD in 484 pupils using two criteria: DSM-IV and neuropsychological criteria. The results showed that the incidence of ADHD using the DSM-IV criterion and the neuropsychological criterion were 18% and 3.5%, respectively [10]. Shabani et al. conducted another research wherein 428 elementary pupils were studied. According to the findings of this study, 4.9% of students (or 21 persons) have ADHD [11].

According to a survey conducted by Adonna et al., 8.7% of Nigerian students aged 7 to 12 had ADHD. In addition, the prevalence of attention-deficit subtypes was determined to be 4.9%, hyperactivity/impulsivity subtype 1.2%, and hyperactivity and attention deficit subtype 2.6% in this study [12]. According to research by Mahala et al., 18.1% of Tunisian youngsters suffer from ADHD [13]. ADHD was identified as the most common disorder among teenagers in another study by Oes Borg et al., which looked into the prevalence of chronic diseases among adolescents with mental disabilities aged 18 − 12 [14].

Another study on the prevalence of ADHD was undertaken by Maulov et al. A total of 210 Lebanese teenagers between the ages of 11 and 17 were studied. 10.2% of persons have ADHD, according to the findings of this study [15].

There are several inconsistencies across research on the prevalence of ADHD in children and adolescents, which are influenced by a variety of factors including diagnostic criteria variances. As a result, this study was conducted to determine the prevalence of ADHD in children and adolescents based on various diagnostic criteria and age groups.

## Method

This research looked at the prevalence of ADHD in children and adolescents by a systematic review and meta-analysis of cross-sectional data. The technique for carrying out the various stages of this study following the criteria of Guideline [16] was created and implemented.

## Eligibility criteria

In general, the criteria for selecting documents in systematic review and meta-analysis are within the framework of PICOS (Population, Intervention, Comparison, Outcomes, Study) [17]. In this study, the eligibility criteria are based on these conditions. Population: All people under the age of 18 were examined in this study. Exposure: Affected children and adolescents. Outcomes: Because of the differences in diagnostic criteria for ADHD and the prevalence of ADHD in different age and gender subgroups, the prevalence of ADHD was evaluated using DSM-V, DSM-IV, DSM IV-TR, and ICD10 criteria. Type of study: Cross-sectional observational studies were utilized to establish the disease’s prevalence. Other observational studies and methodologies, such as interventional review, were left out of the analysis.

## Data sources and search strategy

The authors reviewed four electronic bibliographic databases, including PubMed, Scopus, WOS, and science direct, as well as any electronic publications printed before October 2020. Mesh terms and keywords used in review studies and previously published studies were utilized to choose the relevant keywords, which were chosen by one of the authors (MSH) and confirmed by the first and second authors. All keywords were selected in English. The search strategy in this study was designed using the terms “prevalence OR outbreak OR Global prevalence AND ADHD OR ADDH OR “Attention Deficit Hyperactivity Disorder”.

## Selection of studies

After using Endnote software to remove duplicate articles, an author (MSH) examined the study’s title and abstract. Excluded studies were those that did not meet the entry and exit criteria. The whole text of the paper was then examined by two writers (MSH and HGH) based on inclusion and exclusion criteria. These experiments were carried out independently and by blinding. Any comments given to affirm or reject the research were re-examined after the reviews were completed at this step. In the case of a disagreement, the study was confirmed using the third author’s (MM) reviews. If the full text of the study was not available, an email or research gate request was submitted to the paper’s lead author.

In this study, the STROBE checklist [17] was used as a tool to measure methodological quality in observational studies. According to the various criteria of this checklist, the title, abstract, introduction, method, and results of observational studies with a minimum score of 0 and a maximum score of 32 were considered separately. Studies with a score less than 16 were considered at high risk for bias. However, none of the studies were omitted due to low-quality or high risk of bias, and all studies were analyzed.

## Data extraction

Information gathering was performed by two authors (MSH and HGH). This data was gathered using the methodology and findings of preliminary research. A form was created to extract information such as the first author’s name, year of publication, country, population examined by gender, diagnostic criteria, and diagnostic instruments. Data were extracted by (MSH) and reviewed by (MM).

### Statistical analysis

The heterogeneity of the studies was assessed using the I^2^ test. The Egger test and its accompanying Funnel plot were used to analyze the publication bias due to the large number of samples included in the study. Comprehensive Meta-Analysis software (Version 2) was used to examine the data.

## Results

A systematic review and meta-analysis of information on research on the prevalence of ADHD in children and adolescents around the world was conducted in this study, with no time constraint until 2020 and following PRISMA principles. 1837 potentially related articles were found and transferred to the information management software based on an initial database search (EndNote). A total of 1837 studies were found, with 170 duplicate studies being eliminated. Out of 1667 papers, 1442 were eliminated during the screening phase after titles and abstracts were examined for inclusion and exclusion criteria. 164 articles were removed from the remaining 225 studies during the competency evaluation phase due to irrelevance after analyzing the full text of the article based on inclusion and exclusion criteria. All 61 articles were approved in the qualitative evaluation stage after reading the complete text of the article and calculating the STROBE score (Table 1).

**Table 1:** Information table of studies entered into the systematic review

First author	Year	Country	Assessments	Range of age	Participants	Patient
Abdekhodaie, Zahra [18]	2012	Iran	DSM-IV-TR	5–6	1083	133
Aboul-ata, Mohammad A. [19]	2015	Egypt	DSM-V	6–12 13–14	371 19	68 6
Adewuya, A. O. [12]	2007	Nigeria	DSM-IV	6–12	1112	97
adshaw, L. G. [20]	2017	Qatar	DSM-IV	6–12 13–19	2613 1876	276 97
Afeti, K. [21]	2017	Ghana	DSM-IV	6–12	400	51
Al Azzam, Manar [22]	2017	Jordan		6–12	480	195
Alqahtani, Mohammed MJ [23]	2010	Saudi Arabia	DSM-IV	7–9	708	40
Ambuabunos, EA [24]	2011	Nigeria	DSM-IV	6–12	1473	112
Amiri, S. [25]	2019	Iran	DSM-V	6–9 15–19	298 327	40 8
Anokye, R. [26]	2020	Ghana	DSM-IV	6–9	794	32
Baumgaertel, Anna [27]	1995	Germany	DSM-III DSM-III-R DSM-IV	5–12 5–12 5–12	1077 1077 1077	103 117 192
Bozkurt, Özlem Hekim [28]	2017	Turkey	DSM-IV	6–11	569	72
Bronsard, Guillaume [29]	2011	France	DSM-III-R	13–17	183	7
Canals, Josefa [30]	2018	Spain	DSM-IV	3–6	516	28
Catherine, T. G. Golden [31]	2019	India	DSM-V	8–11	2453	286
Cuffe, Steven P. [32]	2005	America	DSM-IV	4–8 14–17	3557 2819	81 67
de la Barra, Flora Eloísa [33]	2013	Chili	DSM-IV	4–11 12–18	824 734	197 39
Dodangi, N. [34]	2014	Iran	DSM-IV TR	6–11 12–18	145 226	21 23
Egbochuku, EO [35]	2007	Nigeria	DSM-IV	6–12	1384	111
El-Nemr, Fathia Mohamed [36]	2015	Egypt	DSM-IV	5–12	600	118
Erşan, E Erdal [37]	2004	Turkey	DSM-IV	6–12 13–15	1169 256	107 8
fAlyahri, A. [38]	2008	Yemen	DSM-IV	7–10	1210	16
Farahat, Taghreed [39]	2014	Egypt	DSM-IV	6–12	1362	94
Farbstein, Ilana [40]	2014	Israel	DSm-IV, ICD-10	14–17	957	26
Feiz, Pa [41]	2013	Tehran	DSM-IV	6–7	1000	105
Fleitlich-Bilyk, Bacy [42]	2004	Brazil	DSM-IV	7–10	627	17
Ford, T. [43]	2003	England	DSM-IV and ICD-10	5–12 13–15	7814 2624	178 55
Froehlich, Tanya E. T. E. [44]	2007	America	DSM-IV	8–11 12–15	1160 1922	119 103
Gallardo-Saavedra, G. A. [45]	2019	Mexico	DSM-IV	6–8	2832	458
Gau, Susan SF [46]	2005	Taiwan	DSM-IV	13–15	3156	179
Ghosh, Prosenjit [47]	2018	India	DSM-5	6–11	300	38
Gomez, Rapson [48]	2011	Malaysia	DSM-IV	6–12	934	15
Guardiola, A. [10]	2000	Brazil	DSM-IV	6–7	484	87
Gul, N. [49]	2010	Turkey	DSM-IV	6–12	1126	97
Heiervang, E. [50]	2007	Norway	DSM-IV ICD-10	7–9 7–9	9155 9155	16 13
Hs, Ramya [51]	2017	India	DSM-5	5–12	3120	59
Huang, Yanhong [52]	2017	China	DSM-5	7–12	2959	175
Lecendreux, M. [53]	2011	America	DSM-IV	6–12	1012	36
Leib, Shiran [54]	2020	Israel	DSM-IV	13–19	84	24
Leung, Patrick W. L. [55]	2008	China	DSM-IV	ADO	541	24
Maalouf, Fadi T. [15]	2016	Lebanon	DSM-IV and ICD-10	11–12 13–17	177 339	17 35
Mhalla, Ahmed [56]	2018	Tunis	DSM-IV	ADO	447	81
Michanie, C. [57]	2007	Argentina	DSM III-R	6–12	300	27
Mohammadi, Mohammad-Reza [58]	2019	Iran	DSM-IV TR	6–9 15–18	10,466 9409	562 210
Nafi, Omar [59]	2016	Jordan	DSM-IV	6–12	4374	273
Oeseburg, Barth [14]	2010	Netherlands		12–18	1083	229
Pham, Hoai Danh [60]	2015	Vietnam	DSM-IV	5–10	471	31
Pillai, Aravind [61]	2008	India	DSM-IV and ICD-10	12–16	2048	4
Rohde, L. A. [62]	1999	Brazil	DSM-IV	12–14	191	23
Sadolahi, A. [63]	2019	Iran	DSM-5	6–9	806	359
Safavi, Parvin [64]	2016	Iran	DSM-IV	6–12	631	109
Sánchez, Emelyn Y. [65]	2011	Panama	DSM-IV-TR	children	229	17
Shen, Yan-Mei [66]	2018	China	DSM-IV	6–11 12–16	7689 9382	438 409
Talaei, A. [67]	2010	Iran	DSM-IV	7–9	714	109
Umar, M. U. U. [68]	2018	Nigeria	DSM-IV	11–19	487	43
Venkata, Jyothsna Akam [69]	2013	India	DSM-5	6–11	770	72
Vreugdenhil, Coby [70]	2004	Netherlands	DISC-IV	12–18	198	8
Wamithi, Susan [71]	2015	Kenya	DSM-IV	6–12	240	15
Wamulugwa, J. [72]	2017	Uganda	DSM-IV-TR	4–10 10–18	146 186	7 32
Wichstrøm, Lars [73]	2012	Norway	DSM-IV	children	2475	47
Zwirs, Barbara W. C. [74]	2007	Netherlands	DSM-IV	6–10	2041	389

## General prevalence of hyperactivity in children (under 12 years)

**Fig. 1:**
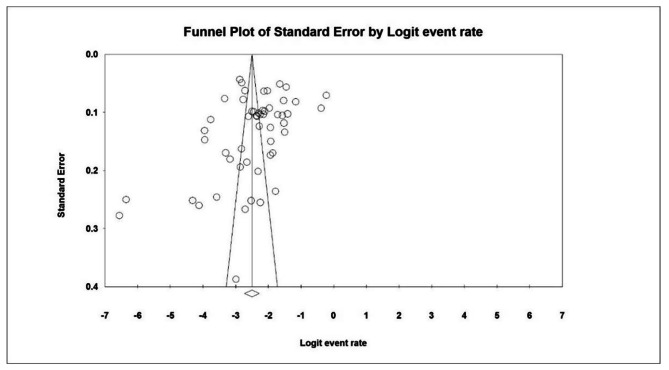
A funnel plot demonstrating the prevalence of hyperactivity in children under the age of 12 years

Based on the I^2^ test results (98.6) and the heterogeneity of the chosen research, a stochastic effects model was utilized to combine studies and estimate overall prevalence. Study heterogeneity can be caused by differences in sample size, sampling error, research year, and study location. The funnel diagram and Egger test at a significance level of 0.05 revealed that there was no publication bias in the distribution of data in the current investigation (P = 0.107). (Fig. 1).

**Fig. 2:**
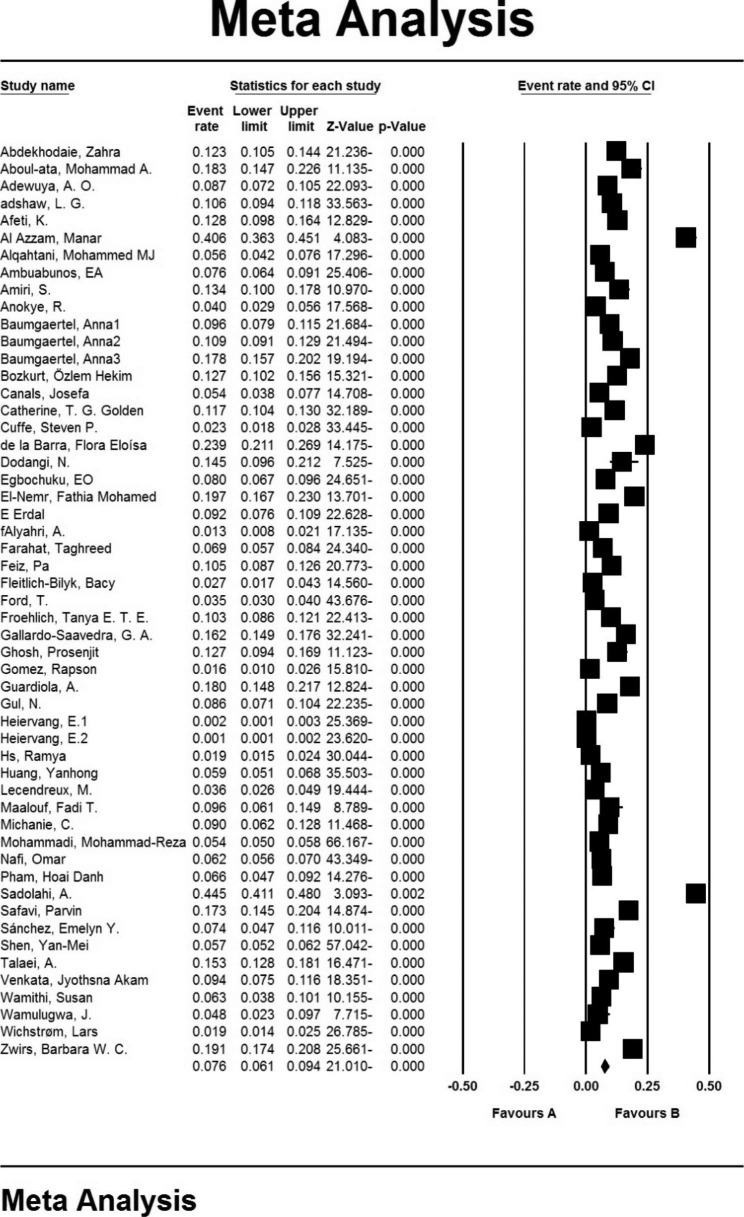
Forest plot, the prevalence of hyperactivity in children under the age of 12 years old

ADHD prevalence was found to be 7.6% (95% confidence interval: 6.1–9.4%) in 53 studies with a total sample size of 96,904 participants who looked at children under the age of 12. The shape of the Forrest plot depicts the overall prevalence in the studies under consideration, with the midpoint of each line segment representing the prevalence in each study and the shape of a rhombus indicating population prevalence for the entire research (Fig. 2).

Based on the results of Table 2 and the methods of diagnosing hyperactivity in children under the age of 12, the DSM-IV‌ diagnostic criterion of ADHD prevalence was 7.7% (95% confidence interval: 6-9.9%).

Other criteria used to determine the prevalence of ADHD in children were DSM-IV-TR, DSM-III, DSM-V, DSM-IV, and ICD10, which were: 8.3% (95% confidence interval: 4.9–13.9%); 10.1% (95%confidence interval: 9-11.3%); 11.3% (95% confidence interval: 5.3–22.2%); and 4.4% (95%confidence interval: 2.4–8.2%), respectively (Table 2).

**Table 2:** Prevalence of hyperactivity in children under 12 years of age based on diagnostic criteria

Diagnostic criteria	N	Sample size	I^2^	Egger test	Prevalence (95%CI)
DSM-IV	33	56,186	98.5	0.052	7.7 (95%CI: 6-9.9)
DSM-IV TR	5	12,069	95.7	0.390	8.3 (95%CI: 4.9–13.6)
DSM-III	3	2454	0	0.572	10.1 (95%CI: 9-11.3)
DSM-IV and ICD-10	3	5963	89.1	0.664	4.4 (95%CI: 2.4–8.2)
DSM-V	8	11,077	99.2	0.509	11.3 (95%CI: 5.3–22.2)

The prevalence of ADHD was also studied in Table 3. According to the findings of 24 studies on ADHD in children under the age of 12, the prevalence of the Inattentive subtype was 33.2% (95% confidence interval: 27.6–39.3%), Hyperactive impulsive subtype was 30.3% (95% confidence interval: 23.8.37.7%), and Combined ADHD was 31.4% (95% confidence interval: 24.6.39.1%) (Table 3).

**Table 3:** Prevalence of hyperactivity in children under 12 years by type of hyperactivity

Diagnostic criteria	N	Sample size	I^2^	Egger test	Prevalence (95%CI)
Inattentive subtype	24	3040	90.5	0.052	33.2 (95%CI: 27.6–39.3)
Hyperactive impulsive subtype	24	3040	93.3	0.390	30.3 (95%CI: 23.8–37.7)
Combined	24	3040	93.1	0.572	31.4 (95%CI: 24.6–39.1)

## General prevalence of hyperactivity in adolescents (12–18 years)

**Fig. 3:**
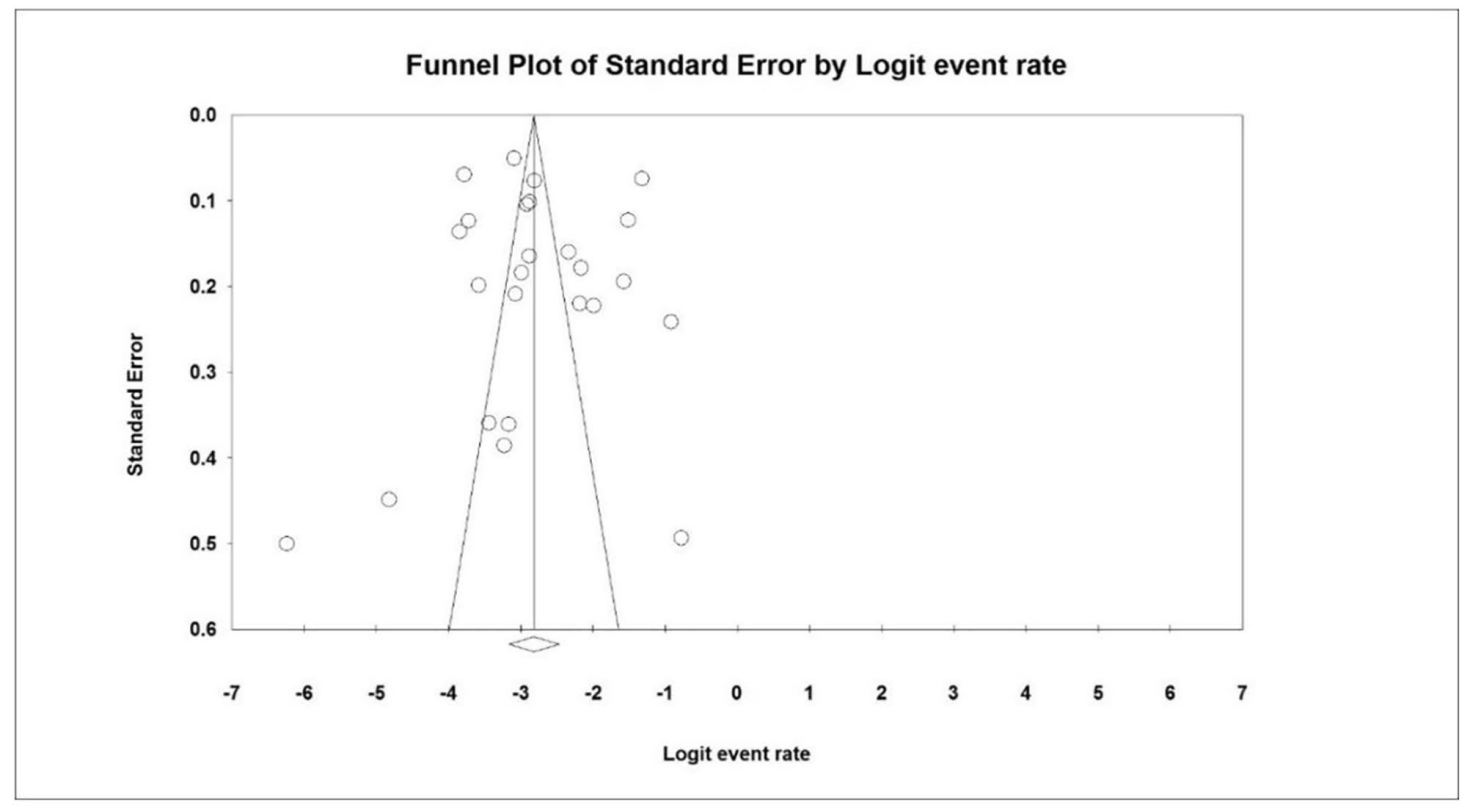
A funnel plot demonstrating the prevalence of hyperactivity in children aged 12 to 18 years old

A random-effects model was used to integrate the studies and estimate the shared prevalence based on the test results (I^2^: 97.8) and the heterogeneity of the selected research. Differences in sample size, sampling error, year, or study location can all contribute to the study heterogeneity The possibility of bias in the dissemination of data was determined using a funnel diagram and the Egger test at a significance level of 0.05 in the current study (P = 0.774). (Fig. 3).

**Fig. 4:**
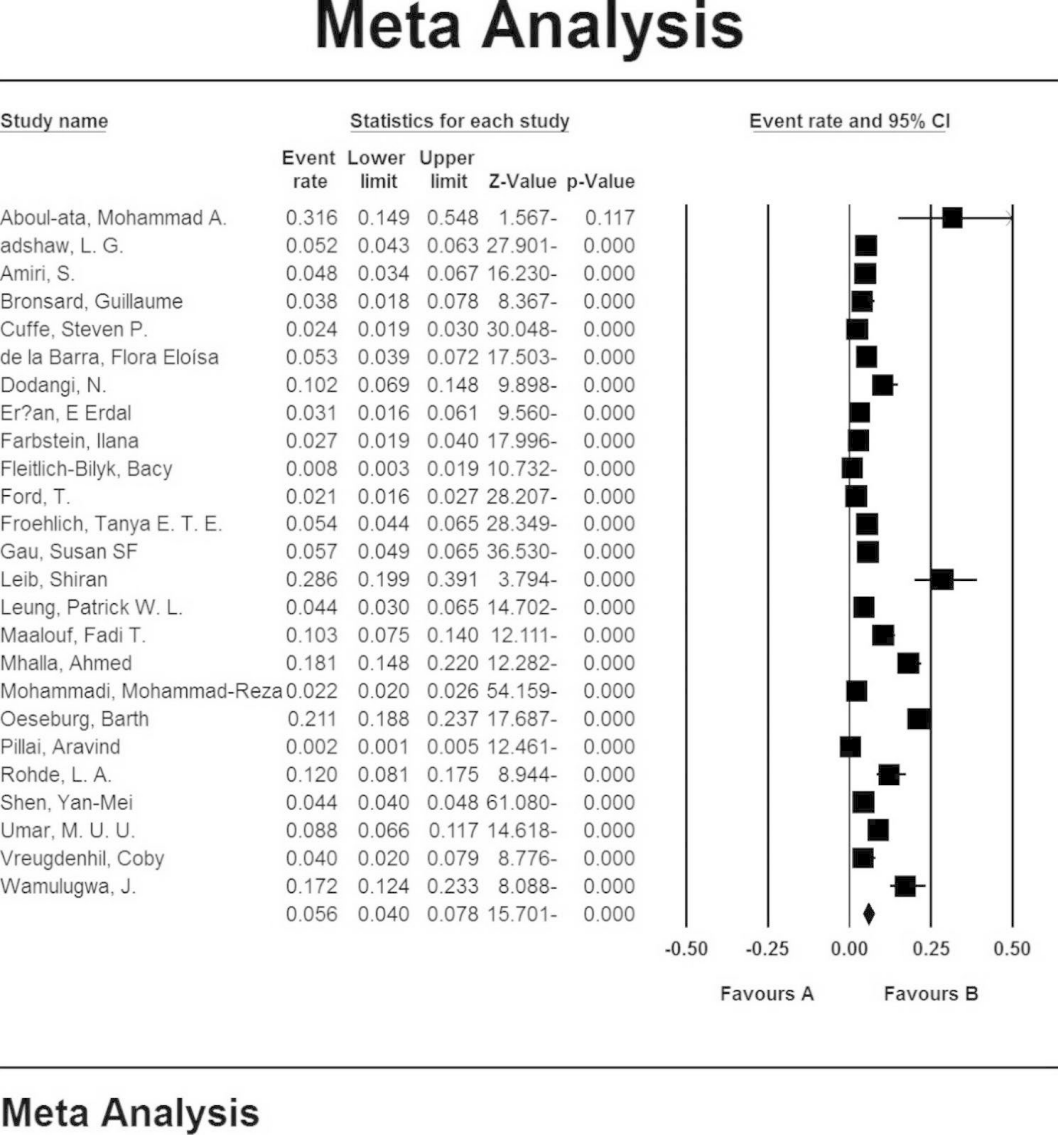
Overall Hyperactivity prevalence in adolescents aged 12 to 18 years

In 25 studies involving teenagers aged 12 to 18, the prevalence of ADHD was determined to be 5.6% (95% confidence interval: 4-7.8%). The total prevalence in the studies examined is depicted in the Forrest plot, with the midpoint of each segment showing the prevalence in each study and the rhombus shape indicating the general population prevalence (Fig. 4).

Using the DSM-IV diagnostic criterion and based on the results of Table 4 and the methods of detecting hyperactivity in children aged 12–18 years, the ADHD prevalence was 7.1% (95% confidence interval: 4.9–10.1%). Other criteria used to determine the prevalence of ADHD in children included DSM-IV-TR, DSM-V, DSM-IV, and ICD10 which reported to be: 7.5% (95% confidence interval: 1.7–15.2%), 12.7% (95% confidence interval: 6.7–19.1%), and 1.7% (95% confidence interval: 1.1–4.5%), respectively (Table 4).

**Table 4:** Hyperactivity prevalence in youth aged 12 to 18 years old depending on diagnostic criteria

Diagnostic criteria	N	Sample size	I^2^	Egger test	Prevalence (95%CI)
DSM-IV	14	23,170	97.5	0.537	7.1 (95%CI: 4.9–10.1)
DSM-IV TR	3	9821	98.6	0.174	7.5 (95%CI: 1.7–15.2)
DSM-V	2	665	94.3	-	12.7 (95%CI: 6.7–19.1)
DSM-IV and ICD-10	5	6592	96.04	0.406	1.7 (95%CI: 1.1–4.5)

Table 5 looked at the prevalence of different kinds of ADHD. According to the findings of five studies involving children aged 12 to 18, the prevalence of Inattentive Subtype was 37.3% (95% confidence interval: 30–45.4%), Hyperactive-Impulsive Subtype was 23.1% (95% confidence interval: 13.2- 38.2%), and Combined ADHD was 31.1% (95% confidence interval: 15.6 -42.3%) (Table 5).

**Table 5:** Prevalence of hyperactivity in children 18 − 12 years according to the type of hyperactivity

Diagnostic criteria	N	Sample size	I^2^	Egger test	Prevalence (95%CI)
Inattentive subtype	5	151	0	0.172	37.3 (95%CI: 30-45.4)
Hyperactive impulsive subtype	5	151	80.7	0.322	23.1 (95%CI: 13.2–38.2)
Combined	5	151	79.3	0.311	31.1 (95%CI: 15.6–42.3)

## Discussion

The objective of this paper was to figure out how prevalent ADHD is in children and adolescents. According to the findings of this study, the prevalence of ADHD in children aged 3 to 12 years is higher than in adolescents aged 12 to 18. It was also shown that the prevalence of this condition varies depending on the diagnostic criteria used. According to studies, the prevalence of ADHD in children and adolescents diagnosed using the DSM-V criteria is higher than using alternative diagnostic criteria. The prevalence of several forms of ADHD was also measured in this study, and the results show that the percentage of ADHD-I, ADHD-H, and ADHD-C is nearly equal in children. Adolescents with ADHD-I, on the other hand, had a slightly higher prevalence of the disorder than those with other forms of ADHD.

ADHD is a neurodevelopmental disorder. Excessive motor activity, inattention, and impulsivity are all symptoms of this disease in children and adolescents [5]. According to the findings, the incidence of ADHD is 7.6% in children aged 3 to 12 years and 5.6% in teenagers aged 12 to 18. The findings of this study are nearly identical to those of earlier meta-analysis studies on the prevalence of ADHD. The prevalence of ADHD in children has been estimated to be between 2 and 7% in previous research [75].

Only one study [76] documented the prevalence of ADHD in teenagers other than children, which is consistent with our findings. According to the findings of a 2012 study, the prevalence of ADHD in children aged 6 to 12 years was 11.4%, and in youngsters aged 12 to 18 years was 8%, which was slightly higher than our findings. The discrepancy is understandable because this study only looked at the DSM-IV criterion. Another study, which looked at the incidence of ADHD in African children and adolescents in 2020, discovered that 7.47% of children and adolescents in Africa had the disorder. The prevalence of ADHD is higher in boys than in girls, according to this study [77]. People under the age of 18 were evaluated in another meta-analysis study conducted in Spain. The prevalence of ADHD in children and adolescents was 6.6% in this study, and 7% in children (participants under 12 years old), which is similar to the current study [78]. Another study looked at the prevalence of ADHD in Chinese children and youths, finding that 5.74% of children and 6.72% of children and adolescents had ADHD [79].

Another analysis carried out in this study was to determine the prevalence of ADHD using various diagnostic criteria. The prevalence of ADHD was higher when using the DSM-V diagnostic criterion than when using other criteria, according to the findings. The DSM-V diagnostic criteria were very similar to the DSM-IV. However, the prevalence and disorder definitions in DSM-V differ from those in DSM-IV. Previously, the spread of the disease was determined by reviewing the symptoms of the disorder, which in the DSM-V is defined as the presence of two or more symptoms of the stated criteria. In light of these circumstances, the rise in the number of patients diagnosed with ADHD based on DSM-V diagnostic criteria is reasonable [80].

According to the results obtained in this study, at adult ages and as people get older ADHD appears to be less common. However, because no follow-up investigations have been conducted, this issue cannot be definitively addressed. Adults have lower rates of ADHD than children, according to previous research [77].

Song P., et al. research was done as a meta-analysis in 2021. The prevalence of ADHD in adults with persistent (childhood AHDH) and symptomatic (not include childhood ADHD) ADHD was investigated in this study. Around 140 million persons were evaluated for persistent ADHD in this study, with only 2.58% having the disease. The study also looked at symptomatic ADHD in a population of 370 million individuals, with 6.76% of people over the age of 18 having the disorder. On the other hand, the findings of this study demonstrated that when participants’ ages increase in both groups, the prevalence of ADHD decreases, which is consistent with our findings [81]. Another study indicated that the prevalence of ADHD was lower in adults over the age of 18 than in teenagers [76]. Even though the prevalence of ADHD in adults is decreasing, it is nevertheless recognized as a risk factor for issues such as academic failure, workplace challenges, and criminality [75]. Adults with ADHD are more likely to commit crimes. As a result, the number of people in prison with ADHD is on the rise [82]. Furthermore, because the onset of ADHD symptoms in adults is socially problematic, many of these people will be admitted to psychiatric hospitals, affecting the prevalence of ADHD in adults in the general population [82, 83].

## Limitation

One of the limitations of this study is the high heterogeneity of studies in terms of age group. Since the purpose of this study was to measure the prevalence of children and adolescents separately, studies that report the prevalence of ADHD in children and adolescents combined were removed. Furthermore, because this study only looked at cross-sectional papers, it was impossible to track the participants and measure their symptoms over time.

## Conclusion

The findings of this study based on meta-analysis demonstrate the significant prevalence of attention deficit hyperactivity disorder (ADHD)The results of this investigation corroborate prior research and highlight the importance of planning and policy-making in the treatment and control of ADHD in children and adolescents. Future research should look into the prevalence of ADHD in different age groups and how it affects their personal and social lives.

## Electronic supplementary material

Below is the link to the electronic supplementary material.

Supplementary Material 1

## Data Availability

Datasets are available through the corresponding author upon reasonable request.

## Abbreviations

ADHD: Attention-Deficit / Hyperactivity Disorder
ADHD-I: ADHD Inattentive type
ADHD-H: ADHD Hyperactive and Impulsive type
ADHD-C: ADHD Combined type
DSM: Diagnostic and Statistical Manual
MESH: Medical Subject Headings
WoS: Web of Science
PRISMA: Preferred Reporting Items for Systematic Reviews and Meta-Analysis
STROBE: Strengthening the reporting of observational studies in epidemiology for cross-sectional study
