# Supplementary material for: The global prevalence of ADHD in children and adolescents: a systematic review and meta-analysis
Source: Ital J Pediatr. 2023 Apr 20;49:48. doi: 10.1186/s13052-023-01456-1 (PMC10120242; doi:10.1186/s13052-023-01456-1)
Supplement: Supplementary file 1 — Supplementary Material 1 [file 13052_2023_1456_MOESM1_ESM.docx]

| **Reviewer #1** | **Respond** |
| --- | --- |
| -the introduction is quite general and does not give enough concrete information. My suggestion is, that it would me more clear if authors explain some basics at the beginning: what is the definition of ADHD; what are the main symptoms. Diagnostic criteria should be explained, and why they are not uniform. Few words about how the criteria have changed recently. Explain differences in ADHD manifestation in boys and girls. Add information about different forms of the disease. All these information is needed to understand results of the meta-analysis. | Added  Page 2, para 6  Page 3, para 2-4 |
| -Systematic revive and meta-analysis on this subject has already been published, (Sayal et al. 2018, Willcut 2012). The authors should explain more clearly, why they have decided to pick up the subject once again | This study was conducted in 2012 and we have updated the study to 2020 and analyzed the subgroup based on diagnostic methods, based on the type of hyperactivity and separately in children and adolescents, which provides reliable and up-to-date information for Use provides researchers. |
| -As for the results, the review would profit by adding analysis on occurrence of ADHD in different sex and comparing connection of particular types of the disease with the sex of patients | Unfortunately, gender information was not reported in all studies, and we could not perform an analysis based on gender, but we emphasized on the diagnostic methods and type of hyperactivity in children and adolescents. |
| -page 3, line 60 : "…the prognosis of the disease is poor [4]. During the developmental process, all children gain the ability to focus, manage activity, and control motivation.]. I do not understand, what do the authors mean. In fact, these two sentences seem to be contradicting. The fact that children gain ability to focus etc. makes the prognosis good, not poor in my opinion. | The second sentence was deleted.  Page 3, para 1 |
| -page 4, lines 13-18: risk factors of ADHD mentioned in the manuscript: "overweight, obesity, and smoking, as well as maternal smoking exposure" - I believe it was a mistake. As far as I know, all these factors refer to maternal exposure during pregnancy. It should be written clearly. | Corrected  Page 3, para 2 |
| -methodology- the methodology is clearly described in the text. So, in my opinion, the Figure 1., with the same information repeated, is redundant. | Figure 1 was removed based on your comment  Page 5, para 5 |
| -there are some abbreviations in the text, that are not explained (for example: ADHA-I, ADHD -H, ADHD-C, DSM, etc.) All the abbreviations should be explained in the text, or the list of abbreviations should be added. | Added  Page 15, para 4 |
| -conclusions: "As a result, ADHD is one of the most frequent childhood and adolescent disorders" In fact this is not a conclusion from this study. To make such conclusion, there should be comparison to prevalence of other disease in childhood. | Corrected  Page 15, para 3 |
| - Finally, and a minor point, this manuscript requires grammatical editing as there are many errors. | The text of the article was reviewed by the native editor and the track changes file is sent to the attachment. |
